# Classifying Normal and Abnormal Status Based on Video Recordings of Epileptic Patients

**DOI:** 10.1155/2014/459636

**Published:** 2014-04-08

**Authors:** Jing Li, Xiantong Zhen, Xianzeng Liu, Gaoxiang Ouyang

**Affiliations:** ^1^Department of Electrical and Automatic Engineering, School of Information Engineering, Nanchang University, Nanchang 330031, China; ^2^Department of Medical Biophysics, University of Western Ontario, Room E5-137, SJHC, 268 Grosvenor Street, London, ON, Canada N6A 4V2; ^3^The Comprehensive Epilepsy Center, Departments of Neurology and Neurosurgery, Peking University People's Hospital, Beijing 100044, China; ^4^State Key Laboratory of Cognitive Neuroscience and Learning & IDG/McGovern Institute for Brain Research, Beijing Normal University, Beijing 100875, China; ^5^Center for Collaboration and Innovation in Brain and Learning Sciences, Beijing Normal University, Beijing 100875, China

## Abstract

Based on video recordings of the movement of the patients with epilepsy, this paper proposed a human action recognition scheme to detect distinct motion patterns and to distinguish the normal status from the abnormal status of epileptic patients. The scheme first extracts local features and holistic features, which are complementary to each other. Afterwards, a support vector machine is applied to classification. Based on the experimental results, this scheme obtains a satisfactory classification result and provides a fundamental analysis towards the human-robot interaction with socially assistive robots in caring the patients with epilepsy (or other patients with brain disorders) in order to protect them from injury.

## 1. Introduction

Epilepsy, one of the most common neurologic disorders, is a chronic disease of brain sudden paradoxical discharge of cortical neurons. It is characterized by the spontaneous and unforeseeable occurrence of seizures [1] with transient signs and/or symptoms due to abnormal, excessive, or synchronous neuronal activity in the brain [2]. It is often accompanied with disturbances in behaviour, short-term brain dysfunction, and cognitive impairment. According to the World Health Organization, the incidence of epilepsy has affected more than 50 million individuals worldwide—about 0.6–1% of the world's population. Because patients with epilepsy have poor ability of independent living, they have lower rates of employment and marriage than others. This not only affects the patients themselves, but also causes fear and inconvenience to their family.

Since the first robot was created in the 1960s, robots have been increasingly used in industrial and entertainment, and more recently the research on socially assistive robots (SARs) for domestic use has received much attention. A socially assistive robot is an intelligent system that is capable of providing assistance for healthy adults or enhancing existing care for persons with cognitive disabilities/impairment, for example, stroke, Alzheimer, and autism spectrum disorder. The question then arises: can socially assistive robots replace nurses or patients' family members for monitoring and caring the epilepsy patients, that is, sounding the alarm, or taking some effective actions to alleviate seizure symptoms?

To address this, human-robot interaction (HRI) [3, 4] is an important issue. Based on the information collected from multiple sensors, HRI works toward smooth interactions between a human user and a socially assistive robot via the use of speech, vision, haptic control, and so forth, for implementing a task. It is a broad area, including a wide variety of research topics, that is, robotics, computer vision, human-computer interaction, modern artificial intelligent, natural language processing, and cognitive science. In the viewpoint of computer vision, the aim of HRI is to understand the patients' behaviors. The first thing is that the robot needs to analyze their motions through action recognition to determine whether the patients are at the seizure status. Otherwise, it may generate unnecessary interaction between the robots and the patients with epilepsy.

According to the International League Against Epilepsy (ILAE), seizure types are organized according to whether the source of the seizure within the brain is localized (partial or focal onset seizures) or distributed (generalized seizures) [5]. Partial seizures, having a focal origin, are further divided based on the extent to which consciousness is affected (simple partial seizures and complex partial seizures) [6]. Generalized seizures affect both cerebral hemispheres (sides of the brain) from the beginning of seizures, such as absence seizures [7] which are short in duration (typically lasting from a few seconds up to around a minute) and may recur over 100 times a day [8]. The seizures may bring the patients with sudden accidents and injuries. Moreover, the sudden and abrupt seizures can appear at any age and may cause serious problems to the patients' body, mind, and intelligence with long-term repeated seizure onset. This supports the importance of detecting seizures as early as possible such that clinicians can prescribe necessary medication for the patients to stop the progression of the chronic disease.

Initially, epilepsy is diagnosed by experienced experts via observing patients' actions, behavioral changes, and mental health history in the family. However, it is not practical in clinical use due to high cost of the manpower and financial resources. During the past few decades, it was confirmed that electroencephalogram (EEG) signals, recording the spontaneous brain electrical activity by means of electrodes located on the scalp, can provide evidence for the existence of a preseizure phase in partial epilepsy [9, 10]. In this paper, we focus on the research of epilepsy and investigate whether the detection/classification of seizure status of patients with epilepsy can be explored by analyzing the movement of epileptics at the video level through human action recognition. As the absence or anomalies of such movement is a highly predictive indicator for epilepsy, accurate classification about the patients' status through video recordings is a fundamental step in detecting different seizure status in the epilepsy. Here, we use computer vision-based techniques to extract the information of movement from video recordings of patients.

Human action recognition [11, 12] is one of the most active topics in computer vision and has been widely applied in video surveillance, video annotation, and retrieval. Current action recognition systems are mainly based on local and holistic representations. Local representations [12] sparsely detect spatiotemporal interest points (STIPs) and have dominated in human action recognition due to their attractive advantages, such as being less sensitive to partial occlusions and clutter and requiring no background subtraction or target tracking as in holistic representations. Nevertheless, local methods suffer from some limitations, one of which is the inability to capture adequate spatial and temporal structure information of actions. On the other hand, holistic representations [13] directly extract spatiotemporal features from raw video sequences and are able to provide entire spatial and temporal structural information of human actions in a sequence. However, they are highly sensitive to partial occlusions and background variations and often require computationally expensive preprocessing steps such as background subtraction, segmentation, and tracking.

To this end, in the task of recognizing normal and abnormal status of epileptics, we propose a simple classification scheme on video recordings by combining local representation with holistic representation, which is able to deal with their shortcomings while integrating their merits. For local representation, we use 3D Gabor filters [14, 15] as they are biologically relevant to human image understanding and recognition. Afterwards, holistic representation is obtained by applying gist features [16] over each filtered volume. Finally, the classification is implemented by a support vector machine (SVM) [17, 18].

To determine whether an epileptic is at the seizure status, the scheme is implemented on the video recordings of epileptic patients at different ages. The movement of affected patients is characterized by more abrupt motion direction changes with periods of no movement. Moreover, the classification task entails lots of challenges: (1) different types of actions: epilepsy appears in different actions, for example, abruptly falling down and continuously vibrating; (2) multiple persons: some video recordings not only contain the patient himself/herself, but also contain the family members or nurses; (3) turning on and turning off the lights in the ward lead to different lighting conditions; and (4) the persons in the videos sometimes wear different-color clothes. Due to the above-mentioned difficulties, the literature that addresses the epilepsy area using video data is limited. Although we only obtain primary results, it is the first time that only video recordings are analyzed for the classification problem in epilepsy, aiming at shedding the light for future research on the prediction of seizure onset. The rest of the paper is organized as follows. In Section 2, we present the newly proposed scheme in detail, including feature extraction based on 3D Gabor features and gist features. In Section 3, we describe the video dataset applied in evaluating the performance of the scheme and report the experimental results.  Section 4 concludes the paper and points out some future works.

## 2. Methods

**Figure 1:**
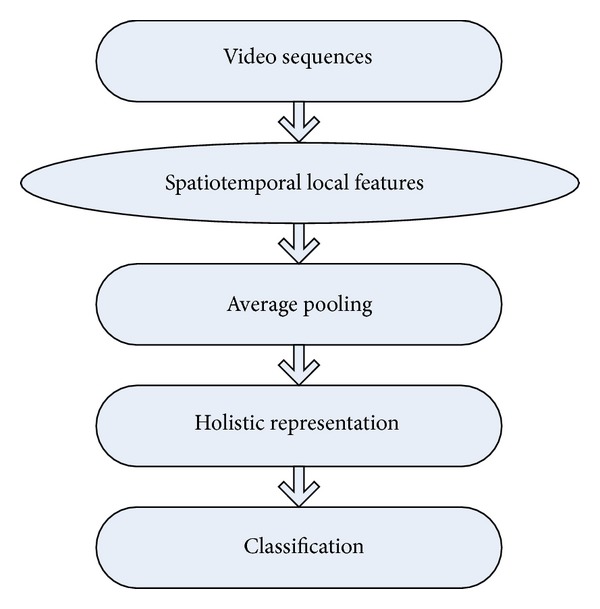
The proposed scheme.

The proposed scheme (as shown in Figure 1) consists of the following two main steps: (i) feature extraction by 3D Gabor features and gist features and (ii) classification by SVM. Each step will be described in detail in the next subsections.

### 2.1. 3D Gabor Filters

Research findings from cognitive psychology and psychophysics suggest that Gabor filters [14, 15] based on image decomposition are biologically relevant to human image understanding and recognition. Consequently, Gabor filters are appropriate for orientation information extraction within a purely computer vision context.

Here, we apply a bank of 3D Gabor filters with one scale and four orientations to localizing salient features in spatiotemporal dimensions, making a total of four Gabor functions. In a 3D space, Gabor filters are defined as

(1) G(x,y,t)=exp⁡(−(X22σx+Y22σy+T22σt)) ×cos⁡(2πλxX)cos⁡(2πλyY)

with

**Figure 2:**
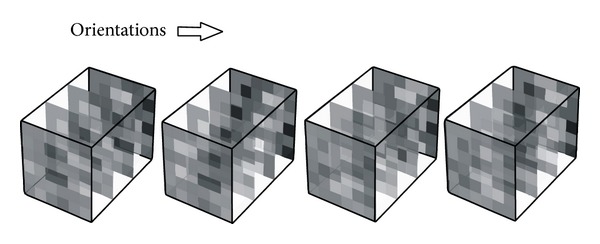
3D Gabor filters on intensity volume.

(2) (XYT)=(1000cos⁡(θ)−sin(θ)0sin(θ)cos⁡(θ)) ×(cos⁡(ω)0sin(ω)010−sin(ω)0cos⁡(ω))(xyt),

where *θ* and *ω* are the spatial and temporal orientations.  Figure 2 shows the 3D Gabor filters on intensity volume in horizontal direction. Since Gabor filters are differential algorithms, the extracted visual features are robust to illumination changes.

### 2.2. Spatiotemporal Gist Feature Extraction

To make compact representation and achieve invariance to small shifts in position and changes in lighting conditions, we use average pooling [19, 20] over the filtered volumes to extract gist features. Similar to the gist feature extraction in scene recognition [16], a gist feature is generated from each filter volume by dividing the volume into a 4 × 4 × 4 grid and then averaging the responses of pixels within each spatiotemporal subregion, resulting in a 256-dimensional feature vector. In this way, the extracted gist features can preserve discriminative information and are tolerant to spatial and temporal shifts and insensitive to noise.

### 2.3. Classification

**Figure 3:**
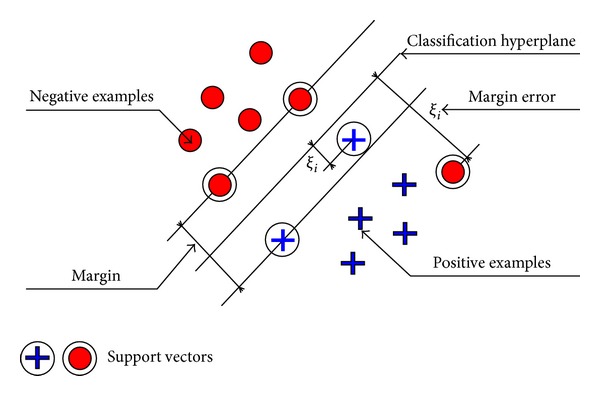
An SVM maximizes the margin between positive examples and negative examples.

A support vector machine (SVM) [17, 18] is a binary classifier, which maximizes the margin between positive examples and negative examples, as shown in Figure 3. Because of its good generalization ability and no requirement for prior knowledge about the data, it has been universally utilized as one of the most popular classifiers in various research areas, for example, face recognition, texture classification, content-based image retrieval (CBIR), and so forth.

Hard-margin SVM and soft-margin SVM are two different forms of an SVM. On one hand, hard-margin SVM solves a quadratic programming problem to deal with linearly separable data. It is effective and requires no parameters. However, it cannot deal with linearly nonseparable examples. On the other hand, soft-margin SVM, the standard solution of a SVM, allows some misclassifications or outliers by adding a regularization term to handle linearly nonseparable data. The methodology of soft-margin SVM is reviewed as follows.

Consider a problem of classifying a set of linearly separable training examples ${\left\{\left({\vec{x}}_{i},y_{i}\right)\right\}}_{i=1}^{N}$ with ${\vec{x}}_{i}\in {ℜ}^{L}$ and their associated class labels *y*_*i*_ ∈ {+1, −1}, an SVM separates these two classes by a hyperplane

(3) $$\begin{array}{c} {\vec{w}}^{T}\cdot \vec{x}+b=0, \end{array}$$

where $\vec{x}$ is an input vector, $\vec{w}$ is an adaptive weight vector, and the scalar *b* is a bias. The optimal hyperplane, which maximizes the geometric margin $2/||\vec{w}||$ between two classes, can be obtained by

(4) min⁡w→,b,ξ→ ||w→||22+C∑i=1Nξis.t. yi(w→T·x→i+b)≥1−ξi,       1≤i≤N ξ→≥0,

where *C* is a constant determined by cross-validation and $\vec{\xi }={\left[{\vec{\xi }}_{1},{\vec{\xi }}_{2},\ldots ,{\vec{\xi }}_{N}\right]}^{T}$ is the vector of all slack variables to deal with the linearly nonseparable problem by giving each misclassified example an individual penalty. For linearly separable training examples, we can set $\vec{\xi }=0$. By introducing a Lagrange multiplier *α*_*i*_, the Lagrangian is

(5) L(w→,b,ξ→,α→,κ→)=12||w||2+C∑i=1Nξi −∑i=1Nαi(yi(w→T·x→i+b)−1+ξi)−∑i=1Nκiξi,

and the solution is determined by

(6) $$\begin{array}{c} \underset{\vec{\alpha },\vec{\kappa }}{\max}\;\underset{\vec{w},b,\vec{\xi }}{\min}L\left(\vec{w},b,\alpha \right), \end{array}$$

which can be achieved by the Karush-Kuhn-Tucker (KKT) conditions

(7) $$\begin{array}{c} \frac{\partial L}{\partial \vec{w}}=0⟹\vec{w}=\overset{N}{\underset{i=1}{\sum }}\alpha_{i}y_{i}{\vec{x}}_{i},\\ \frac{\partial L}{\partial b}=0⟹{\vec{\alpha }}^{T}\vec{y}=0,\\ \frac{\partial L}{\partial \xi }=0⟹C-\vec{\alpha }-\vec{\kappa }=0. \end{array}$$

Therefore, the parameters $\vec{w}$ and *b* can be obtained using the Wolfe dual problem

(8) max⁡α→ Q(α)=∑i=1Nαi−12∑i,j=1Nαiαjyiyj(x→Ti·x→j)s.t. 0≤αi≤C   α→Ty→=0.

Most of *α*_*i*_ are zeros, and ${\vec{x}}_{i}$ corresponding to *α*_*i*_ > 0 are referred to the support vectors. In Figure 3, they are expressed as the examples close to the decision boundary or at the wrong side of the margin.

In the dual format, data points only appear in the inner product. To solve the nonlinearly separable problem, the data points from the low-dimensional input space *L* are mapped onto a higher dimensional feature space *H* (the Hilbert inner product space) by the replacement

(9) $$\begin{array}{c} {\vec{x}}_{i}\cdot {\vec{x}}_{j}⟶\phi \left({\vec{x}}_{i}\right)\cdot \phi \left({\vec{x}}_{j}\right)=K\left({\vec{x}}_{i},{\vec{x}}_{j}\right), \end{array}$$

where $K\left({\vec{x}}_{i},{\vec{x}}_{j}\right)$ is a kernel function with entries $\phi ({\vec{x}}_{i})$, $\phi ({\vec{x}}_{j})\in {ℜ}^{H}$. A lot of standard kernel functions can be embedded in SVMs, such as linear kernels $K\left({\vec{x}}_{i},{\vec{x}}_{j}\right)={{\vec{x}}^{T}}_{i}\cdot {\vec{x}}_{j}$, polynomial kernels $K\left({\vec{x}}_{i},{\vec{x}}_{j}\right)={\left({\vec{x}}_{i}\cdot {\vec{x}}_{j}+c\right)}^{d}$, Gaussian radial basis function (RBF) $K\left({\vec{x}}_{i},{\vec{x}}_{j}\right)=\exp\{-{||{\vec{x}}_{i}-{\vec{x}}_{j}||}^{2}/2\sigma^{2}\}$. Then, the kernel version of the Wolfe dual problem is

(10) $$\begin{array}{c} Q\left(\alpha \right)=\overset{N}{\underset{i=1}{\sum }}\alpha_{i}-\frac{1}{2}\overset{N}{\underset{i,j=1}{\sum }}\alpha_{i}\alpha_{j}y_{i}y_{j}K\left({\vec{x}}_{i}\cdot {\vec{x}}_{j}\right). \end{array}$$

Finally, for a given kernel function, the SVM classifier is given as

(11) $$\begin{array}{c} F\left(\vec{x}\right)=\mathrm{sgn}\left(f\left(\vec{x}\right)\right), \end{array}$$

where $f(\vec{x})=\sum_{i=1}^{l}\alpha_{i}y_{i}K\left({\vec{x}}_{i},{\vec{x}}_{j}\right)+b$ is the output hyperplane decision function of SVM.

In traditional SVM-based RF algorithms, $f(\vec{x})$ is used for measuring the dissimilarity between the query image and an example image in the database. For a given example, a high $f(\vec{x})$ indicates that it is far away from the decision boundary and thus has high prediction confidence while a low $f(\vec{x})$ shows that it is close to the boundary and its corresponding prediction confidence is low.

## 3. Results and Discussion

In this section, we describe the process of video data acquisition and experimental setup. Afterwards, the experimental results that evaluate the effectiveness of our proposed scheme are reported.

### 3.1. Video Data Acquisition and Experimental Setup

We collected 41 video recordings of 9 epilepsy patients at a resolution of 640 × 480. The videos were recorded with a frame rate of 25 frames/s in the AVI video format. Each patient was lying on a standard hospital bed, and a stationary digital video camera was placed at a distance above the patients to record the movements. This resulted in an experimental setup where a similar camera position was assured for all recordings. Please note that not every video clip recorded the seizure status; that is, some videos were selected to ensure an awake and comfortable state. The original 41 video recordings are of different length, ranging from less than one minute to nearly ten minutes. Considering this, we segment each video into several ones that are no longer than one minute. Afterwards, each of them is converted into the AVI video format at a resolution of 160 × 128. The study protocol had previously been approved by the ethics committee of Peking University People's Hospital and the patients had signed informed consent that their clinical data might be used and published for research purposes.

**Figure 4:**
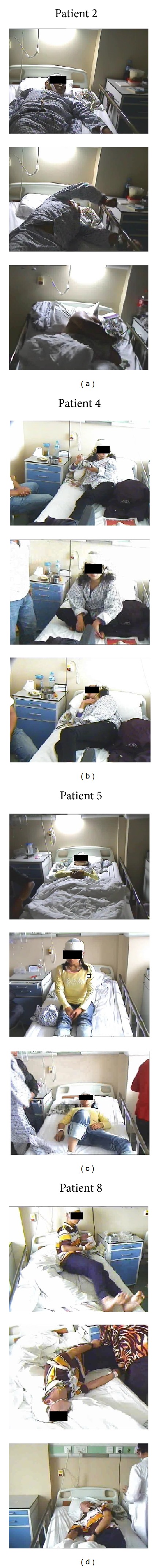
Sample video frames from Patient 2, Patient 4, Patient 5, and Patient 8, respectively.

Figure 4 shows sample video frames from Patient 2, Patient 4, Patient 5, and Patient 8, respectively, where frames in a column belong to the same patient. As we can see, with this dataset, the classification task entails lots of challenges: (1) different types of actions: epilepsy appears in different actions, for example, abruptly falling down, continuously shaking, and so forth; (2) multiple persons: some video recordings not only contain the patient himself/herself, but also contain the family members or nurses; (3) the patients are usually in different lighting conditions; and (4) the persons in the videos sometimes wear different-color clothes. All of the four points mentioned above bring about some difficulty in distinguishing normal status from abnormal status.

To evaluate the classification performance, we manually annotated each video recording with a bounding box to locate the epilepsy child. Moreover, a label, that is, “normal” or “abnormal,” is assigned to each video.

For classification, we make use of an SVM classifier with a linear kernel due to its good generalization ability and efficiency. Classification consists of the training phase and the testing phase. We randomly select half of the video clips for each patient for training and use the rest for testing. This step is conducted for 5 times and the average accuracy is 65.22%. After training the classifier based on the extracted features of the training set, the classifier is trained and able for classifying the examples in the testing set. We define two categories labeled as “normal” and as “abnormal,” which denote different states of the tested epileptics.

Although the accuracy is not very high, but with such a complex video dataset, the classification performance is satisfactory and can serve as a tool for automatically predicting the seizure status of the epilepsy patients. The current video data include epilepsy patients of different ages. An interesting question is how the movement changes with an increasing age of the patients and whether this can be used as a feature value.

## 4. Conclusions

This paper explores whether the normal status and the abnormal status of epileptic patients can be distinguished based on video recordings rather than traditional EEG recordings. Combining local representation and holistic representation, the extracted features are effective for the subsequent classification by an SVM. Our future goal is to capture the characteristic abrupt movements of epileptics by developing new features that are effective in detecting abnormal actions of the epileptic patients.

As pointed in [21], higher frame rates of video recordings could increase the accuracy of the motion estimation and result in a high quality of motion tracking. Another promising option that can be explored in the future would be to collect Kinect videos using RGB-D camera. In this way, depth information of 3D points can be included for motion tracking. What is more, we would collect more video data and classify the actions into different types in our future work. Last, we intend to extend this work into the research of other medical areas, such as autism and Alzheimer.
